# Growth Behavior, Biomass Composition and Fatty Acid Methyl Esters (FAMEs) Production Potential of *Chlamydomonas reinhardtii*, and *Chlorella vulgaris* Cultures

**DOI:** 10.3390/md21080450

**Published:** 2023-08-15

**Authors:** Itzel Y. López-Pacheco, Victoria Guadalupe Ayala-Moreno, Catherinne Arlette Mejia-Melara, José Rodríguez-Rodríguez, Sara P. Cuellar-Bermudez, Reyna Berenice González-González, Karina G. Coronado-Apodaca, Leonardo I. Farfan-Cabrera, Georgia María González-Meza, Hafiz M. N. Iqbal, Roberto Parra-Saldívar

**Affiliations:** 1 Tecnologico de Monterrey, School of Engineering and Sciences, Monterrey 64849, Mexico; a00824134@tec.mx (I.Y.L.-P.); jrr@tec.mx (J.R.-R.); sara.cuellar@tec.mx (S.P.C.-B.); reyna.g@tec.mx (R.B.G.-G.); karina.coronado@tec.mx (K.G.C.-A.); farfanl@tec.mx (L.I.F.-C.); georgia.gonzalez@tec.mx (G.M.G.-M.); 2 Tecnologico de Monterrey, Institute of Advanced Materials for Sustainable Manufacturing, Monterrey 64849, Mexico; 3 Francisco Morazán Department, Escuela Agrícola Panamericana, Zamorano, Km 30 Carretera de Tegucigalpa a Danlí, Valle del Yeguare, Municipio de San Antonio de Oriente, Tegucigalpa 11101, Honduras; victoria04ayala@outlook.com (V.G.A.-M.); kathy.11mejia@gmail.com (C.A.M.-M.)

**Keywords:** microalgae, fatty acid methyl esters, proteins, biorefinery

## Abstract

The production of biomolecules by microalgae has a wide range of applications in the development of various materials and products, such as biodiesel, food supplements, and cosmetics. Microalgae biomass can be produced using waste and in a smaller space than other types of crops (e.g., soja, corn), which shows microalgae’s great potential as a source of biomass. Among the produced biomolecules of greatest interest are carbohydrates, proteins, lipids, and fatty acids. In this study, the production of these biomolecules was determined in two strains of microalgae (*Chlamydomonas reinhardtii* and *Chlorella vulgaris*) when exposed to different concentrations of nitrogen, phosphorus, and sulfur. Results show a significant microalgal growth (3.69 g L^−1^) and carbohydrates (163 mg g^−1^) increase in *C. reinhardtii* under low nitrogen concentration. Also, higher lipids content was produced under low sulfur concentration (246 mg g^−1^). It was observed that sulfur variation could affect in a negative way proteins production in *C. reinhardtii* culture. In the case of *C. vulgaris*, a higher biomass production was obtained in the standard culture medium (1.37 g L^−1^), and under a low-phosphorus condition, *C. vulgaris* produced a higher lipids concentration (248 mg g^−1^). It was observed that a low concentration of nitrogen had a better effect on the accumulation of fatty acid methyl esters (FAMEs) (C16-C18) in both microalgae. These results lead us to visualize the effects that the variation in macronutrients can have on the growth of microalgae and their possible utility for the production of microalgae-based subproducts.

## 1. Introduction

Microalgae biomass can be useful for the production of many materials, including bioplastics, food supplements, biofuels, UV-blockers, antimicrobial bio-compounds, and animal feed, among others [1]. In general, microalgae biomass has been reported to produce and accumulate carbohydrates, proteins, lipids, and fatty acids (FAs) [2], which makes it an interesting source of renewable raw material for a wide range of applications. Moreover, microalgae are considered a sustainable source of materials, since these microorganisms can be applied in bioremediation processes and convert waste into value-added products in a circular economy scheme [3]. For example, microalgae have been employed to reduce CO_2_ emissions via bio-fixation processes because they can capture CO_2_ through the photosynthetic pathway. In this process, microalgae use CO_2_ from the atmosphere or other sources like flue gas streams for biomass production; it has been determined that producing 1 g of microalgae biomass represents the capture of 1.8 g of CO_2_ [4,5,6]. Thus, environmental benefits are also involved with the use of microalgae biomass as feedstock for some processes.

Another bioremediation application is the use of microalgae for wastewater treatment processes. In this respect, researchers have reported the removal of water pollutants, including up to 90% of COD, 100% nitrogen, and 50% phosphorous, producing not only good-quality effluents but also biomass that is able to be employed for the production of biofuels, bioplastic, and food sources, among other applications [7]. For example, *Chlorella* biomass from a swine wastewater phycoremediation process exhibited higher production of lipids—including triacylglycerol—in comparison to that obtained in the standard medium [8]; these lipids can be used in bioplastic production.

Microalgae use depends on its biomass composition in terms of macromolecules (proteins, lipids, and carbohydrates) and active compounds, which in turn depends on microalgae strains and microalgae culture conditions [9]. In the case of carbohydrate content, some microalgae strains can accumulate >25% dry wt of carbohydrates such as *Scenedesmus*, *Dunaliella*, and *Chlorella* (Chlorophyta) [10]. In the case of proteins, *Galdieria sulphuraria* (Rhodophyta, Cyanidiophyceae) can accumulate >60% dry wt [11], while *Chlorella vulgaris* can accumulate 48% dry wt [12]. Regarding lipids, microalgae can produce a yield per hectare that is at least 7 times higher than other oil crops (i.e., soybean, sunflower, corn) [13]. *Chlorella* sp. (Chlorophyta), *Phaeodactylum tricornutum* (Bacillariophyta), and *Botryococcus braunii* (Chlorophyta) can produce >20% dry wt of lipids [14,15].

Triacylglycerols are non-polar lipids formed by three fatty acids esterified with glycerol. They are common molecules for energy storage in microalgae cells and the most common dietary fat for some organisms, such as humans [16]. Different FAs in microalgae have been identified, such as short-chain fatty acids, medium-chain fatty acids, and long-chain fatty acids. Palmitic (C16:0), stearic (C18:0), palmitoleic (C16:1), oleic (C18:1), linoleic (C18:2), and linolenic (C18:3) acids are examples of long-chain fatty acids found in microalgae [17]. Also, polyunsaturated fatty acids (PUFAs) have been found, including docosapentaenoic acid (DPA), eicosapentaenoic acid (EPA), and docosahexaenoic acid (DHA), which are very important compounds in the food industry [18]. Microalgae biomass, especially its FAs content, has demonstrated potential for biodiesel production, and particular varieties can be useful in determining possible biodiesel characteristics [19] but also for bio-based lubricants [20], the latter being more sought and valued in recent decades.

In this manner, several applications exist for microalgae biomass and different biomolecules. For example, carbohydrates can be used to produce bioethanol, biobutanol, biomethane, bioplastics, food supplements, animal feed, and fertilizers [21,22,23]. Proteins can be used in human food, protein supplements [24], and stabilizers for food, cosmetic, and pharmaceutical products [25]. Lipids can be used for bioenergy, since the fatty acids in microalgal biomass produce high-quality biodiesel [26]. Also, lipids can act as surfactants or emulsifiers in cosmetics to provide additional properties. For example, adding PUFAs in cosmetic formulations provides anti-inflammatory and antioxidant properties [27]. Recently, the use of microalgae for the obtention of lubricant oils such as metalwork fluids has been explored. For this application, microalgae should produce long-carbon-chain (C > 16) FAs [28]. Moreover, microalgae cultures have been considered as a viable option for such macromolecules, since their production can be more sustainable [29].

In this context, microalgae biomass can be used to obtain specific compounds according to the desired application. Thus, different strategies have been investigated to obtain higher yields of certain biomolecules in microalgae biomass. For example, some researchers have evaluated pH variations [30], different carbon sources [31], variations in culture medium composition [32], and different photoperiods and light intensity modifications [33]. A simple technique consists of modifying the culture medium by reducing the supply of some macroelements; typically, nitrogen and phosphorus are the elements that are most commonly varied, causing an increment in carbohydrates, proteins, and lipids. This strategy has demonstrated good potential; thus, varying other elements like sulfur should be further investigated (as they are in this article). This study evaluated the growth and biomass composition of two microalgae strains under different macronutrient concentrations. It was desirable to carry out this experimentation because in most articles, these macroelements (nitrogen, phosphorus, and sulfur) do not vary in the same microalga. Therefore, this study shows that the variation in these macroelements can impact the production of macromolecules of interest, which could be interesting for the industrial sector. The total carbohydrate, protein, and lipid production from *Chlamydomonas reinhardtii* and *Chlorella vulgaris* (Chlorophyta) was determined in response to different nitrogen, phosphorus, and sulfur concentrations. Moreover, the FAMEs production of these two microalgae strains was evaluated, which makes this article novel, because there are very few that evaluate the specific production of fatty acids by increasing or reducing the concentration of macroelements in the culture medium. The effect of varying the nitrogen, phosphorus, and sulfur concentrations in the culture medium was analyzed in detail to maximize the biomolecules yield derived from microalgae biomass.

## 2. Results and Discussion

### 2.1. Growth Behavior of Chlamydomonas reinhardtii and Chlorella vulgaris with Different Nitrogen, Phosphorus, and Sulfur Concentrations

**Figure 1:**
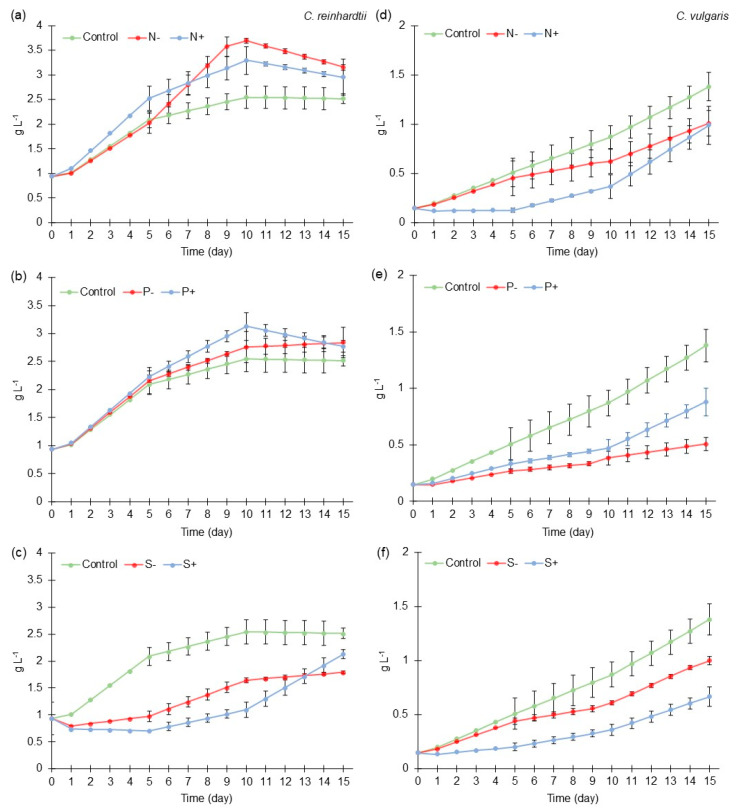
Growth behavior (g L^−1^) of *Chlamydomonas reinhardtii* and *Chlorella vulgaris* at different nitrogen, phosphorus, and sulfur concentrations. (**a**) Cell growth of *Chlamydomonas reinhardtii* grown at different nitrogen concentrations. (**b**) Cell growth of *Chlamydomonas reinhardtii* grown at different phosphorus concentrations. (**c**) Cell growth of *Chlamydomonas reinhardtii* grown at different sulfur concentrations. (**d**) Cell growth of *Chlorella vulgaris* grown at different nitrogen concentrations. (**e**) Cell growth of *Chlorella vulgaris* grown at different phosphorus concentrations. (**f**) Cell growth of *Chlorella vulgaris* grown at different sulfur concentrations. All points were sampled by triplicate. The samples were taken every day.

In standard medium concentration [Control], *C. reinhardtii* showed cell growths of 2.08 g L^−1^, 2.54 g L^−1^, and 2.51 g L^−1^ at the 5th, 10th, and 15th culture days, respectively (Figure 1a–c). These results are comparable to those obtained by Morales-Sánchez et al. (2020) [34], where *Chlamydomonas malina* FT89.6 PG5 (Roscoff Culture Collection No. 2488) (https://roscoff-culture-collection.org/rcc-strain-details/2488, accessed on 10 June 2023) reached approximately 4 g L^−1^ at the 10th culture day. *C. vulgaris* culture showed a better microalgae growth at medium concentration of all macroelements [Control]; it reached 0.86 g L^−1^ of biomass growth at the 10th and 1.37 g L^−1^ at the 15th culture day (Figure 1d–f). These results are similar to those found by Josephine et al. [35], where >1 g L^−1^ was obtained at the 15th culture day of *C. vulgaris*.

*C. reinhardtii* presented a better growth at low nitrogen concentration [N^−^] (3.69 g L^−1^ at 10th day) and at high phosphorus concentration [P^+^] (3.12 g L^−1^ at 10th day) (Figure 1a,b). In this manner, an increment of at least 0.40 g L^−1^ can be obtained by modifying the concentration of nitrogen [N^−^,N^+^] or phosphorous [P^+^] in comparison to the medium concentration [Control] after ten days of cultivation. It was shown that high concentration of nitrogen [N^+^] and sulfur [S^+^] had a noticeable negative effect on *C. vulgaris* growth, where 0.98 g L^−1^ and 0.66 g L^−1^ were obtained at the 15th culture day, respectively. However, at high nitrogen [N^+^] concentration, *C. vulgaris* showed the tendency to increase microalgae growth after the 10th culture day, similar to the low nitrogen [N^−^] concentration curve. These results are related to those found with *C. reinhardtii*, since both microalgae had better growth in low-nitrogen treatments [N^−^]. Also, the inhibition effect on microalgae growth caused by high nitrogen loads in culture medium has been demonstrated [36].

Other studies have reported that low-nitrogen conditions in the culture medium increased cell growth. For instance, *Isochrysis galbana* (Haptophyta, Coccolithophyceae) presented enhanced cell growth at the nitrogen concentration of 144 mg L^−1^ in comparison to the nitrogen concentration of 288 mg L^−1^ [36]. Similarly, *Graesiella emersonii* (formerly *Chlorella emersonii*) (Chlorophyta) microalgae reached a higher cell growth under low-nitrogen conditions than under nitrogen abundance [37].

The phosphorous concentration in the *C. reinhardtii* culture had a strong effect as well; optimal results were obtained under the high concentration [P^+^]. This result is in agreement with those reported by Lovio Fragoso et al. (2019) [38], who cultivated *Chaetoceros muelleri* (Bacillariophyta) under high-phosphorus conditions, showing a slight increase in cell growth. On the other hand, for *C. vulgaris*, it was shown that lower concentration [P^−^] has a more negative effect on microalgae growth than the effect obtained with high concentration [P^+^]. The same effect was reported by Li et al. (2022) [39], who cultivated *Tetradesmus obliquus* (formerly *Scenedesmus obliquus*) (Chlorophyta) under low-phosphorus condition (0.02 mg L^−1^); experiments under low-concentration conditions showed lower values in terms of cell growth when compared to those obtained under high concentrations of phosphorus (2 mg L^−1^) [39]. In this experiment, an exponential phase was not shown in any culture, so they can be maintained for a longer number of days.

Regarding sulfur, *C. reinhardtii* growth was negatively affected by this macronutrient, since 1.79 g L^−1^ and 2.12 g L^−1^ of microalgae biomass were obtained under low [S^−^] and high [S±] sulfur concentrations at the 15th day, respectively (Figure 1c). Also, it was shown that high concentration of sulfur [S^+^] had a noticeable negative effect on *C. vulgaris* growth, which was 0.66 g L^−1^ at the 15th culture day. Sulfur deprivation decreases photosynthetic activity in microalgae; thus, the low results for cell growth could be related to this phenomenon. In the case of high sulfur treatment, Mao et al. (2020) [40] reported a similar growth behavior, in which the variation in sulfur concentration affected microalgae growth during the first days of culture, subsequently increasing cell growth, until adapting a similar behavior to that presented by the control.

Also, it can be shown that at the 10th day of most microalgae cultures [Control, N^−^, N^+^, P^+^, P^−^, S^−^], the exponential phase is reached in *C. reinhardtii* culture. The exponential phase of this microalgae can be reached usually at the 8th or 10th day of culture, even in light stress, salinity stress, or nutrient stress conditions. This behavior could be related to nutrients depletion and less light penetration because of its high cell density [34]. However, this behavior could imply that this microalga can be useful for obtaining large amounts of microalgae biomass in short times.

From ANOVA–Tukey pairwise analysis of *C. reinhardtii* growth, it was determined that at low nitrogen [N^−^] concentration, a higher microalgae biomass was produced in all the experiments at the 10th day of culture (3.69 g L^−1^). In the case of *C. vulgaris* culture, it was determined by the ANOVA–Tukey pairwise analysis that at medium [Control] concentration of macroelements, a higher microalgae biomass was produced in all the experiments at the 15th day of culture (1.37 g L^−1^). These results demonstrate that the growth behavior of microalgae with different nitrogen, phosphorus, and sulfur concentrations depends on the microalgae strain; however, some similar trends were found. In the case of *Chlorella vulgaris*, it can be concluded that the variations made negatively affected cell growth, because the variation was excessive for the culture to adapt in such a short time. However, since the culture did not reach a stationary phase, it was not discarded that in later days the behavior would have changed.

### 2.2. Carbohydrates Content of Chlamydomonas reinhardtii and Chlorella vulgaris Grown with Different Nitrogen, Phosphorus, and Sulfur Concentrations

**Figure 2:**
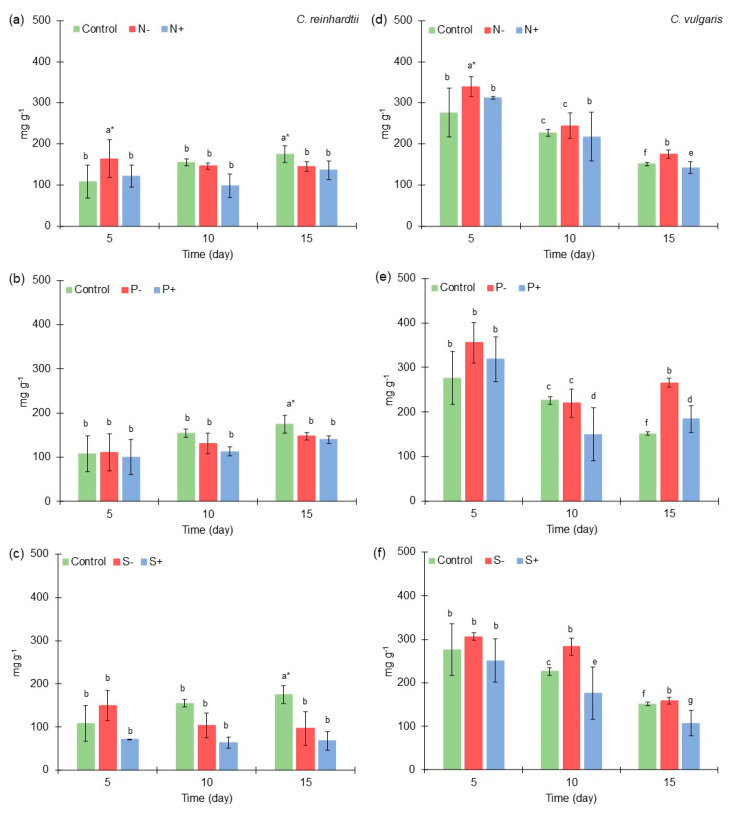
Carbohydrates content (mg g^−1^) of *Chlamydomonas reinhardtii* and *Chlorella vulgaris* grown at different nitrogen, phosphorus, and sulfur concentrations. (**a**) Carbohydrates content of *Chlamydomonas reinhardtii* grown at different nitrogen concentrations. (**b**) Carbohydrates content of *Chlamydomonas reinhardtii* grown at different phosphorus concentrations. (**c**) Carbohydrates content of *Chlamydomonas reinhardtii* grown at different sulfur concentrations. (**d**) Carbohydrates content of *Chlorella vulgaris* grown at different nitrogen concentrations. (**e**) Carbohydrates content of *Chlorella vulgaris* grown at different phosphorus concentrations. (**f**) Carbohydrates content of *Chlorella vulgaris* grown at different sulfur concentrations. The results of the ANOVA–Tukey pairwise analysis carried out for each microalga in all the variations in macroelements also could be observed by different groups expressed by letters (symbol * = express the treatment where a higher production was obtained throughout the experiment). All points were sampled by triplicate. The samples were taken every five days.

*C. reinhardtii* produced a higher concentration of carbohydrates with medium macroelements concentration [Control] in most microalgae cultures (Figure 2a–c), except in the cases of low nitrogen [N^−^] and sulfur [S^−^] concentration at the 5th culture day. Regarding *C. vulgaris*, an improved production of carbohydrates was obtained with lower macroelements concentrations in most of the microalgae cultures (Figure 2d–f).

At medium macroelements concentration [Control], *C. reinhardtii* obtained 108 mg g^−1^, 155 mg g^−1^, and 175 mg g^−1^ of carbohydrates at the 5th, 10th, and 15th culture days, respectively. On the other hand, *C. vulgaris* at medium macroelements concentration obtained 276 mg g^−1^, 226 mg g^−1^, and 151 mg g^−1^ of carbohydrates at the 5th, 10th, and 15th culture days, respectively. These results can be compared to those obtained by Morales-Sánchez et al. (2020) [34], where *Chlamydomonas malina* (Chlorophyta) RCC2488 reached approximately 200 mg g^−1^ at the 10th culture day.

At low nitrogen [N^−^] and sulfur [S^−^] concentration at the 5th culture day in *C. reinhardtii* culture, 163 mg g^−1^ (50% greater than medium concentration) and 149 mg g^−1^ (37% greater than medium concentration) were obtained, respectively. The effect of nitrogen and sulfur deprivation in enhancing the carbohydrate content in microalgae biomass has been reported by different authors and using different microalgae strains. For example, in the case of *C. reinhardtii* CC-124, an increase of up to 4.3-fold was obtained [41], while *Vischeria calaminaris* (formerly *Eustigmatos calaminaris)* (Ochrophyta, Eustigmatophyceae) under nitrogen limitation conditions accumulated >80% of carbohydrates [42]. It is important to note, that the variation in some macroelements affects carbohydrates content in *C. reinhardtii* in a negative way, especially high concentrations of nitrogen, phosphorus, and sulfur.

In the experiments on nitrogen variations in *C. vulgaris* culture, a greater amount of carbohydrates was obtained at low nitrogen concentration [N^−^], where 339 mg g^−1^ (22% greater than medium [Control] concentration), 244 mg g^−1^ (8% greater than control), and 174 mg g^−1^ of carbohydrates (15% greater than control) were obtained at the 5th, 10th, and 15th culture days, respectively. These results can be compared to those obtained by Cheng et al. (2017) [43], where *Chlorella* sp. growing in standard medium had an accumulation of 20–40% dry wt of carbohydrates. In their study, it was found that nitrogen limitation enhances carbohydrates accumulation in *Chlorella* sp. biomass, such as in *Tetradesmus obliquus* (formerly *Scenedesmus obliquus*) (Chlorophyta), where 43% dry wt of carbohydrates was obtained compared to the 25% dry wt reached under standard conditions.

Also, a greater amount of carbohydrates was obtained under low phosphorus concentration [P^−^] in *C. vulgaris* culture, but only on days 5 and 15 of the microalgae culture, when 355 mg g^−1^ (28% greater than control) and 265 mg g^−1^ of carbohydrates (>50% greater than control) were obtained, respectively. It was shown in previous studies that low phosphorus concentrations could enhance the production of carbohydrate. For example, in *Chlorella* spp. culture, >50% of carbohydrates were obtained under phosphorus limitation [44]. Also, similar to this study, the limitation of phosphorus caused a greater accumulation of carbohydrates compared to results obtained under nitrogen limitation conditions [44]. Also, in *Scenedesmus*. LX1 culture, the carbohydrates production increased at least 20% under low-nutrient conditions (nitrogen/phosphorus) compared to that obtained in high-nutrient conditions [45].

At low sulfur concentrations [S^−^] in *C. vulgaris* culture, the same behavior was observed that was obtained with low nitrogen and phosphorus concentration, since 306 mg g^−1^ (10% greater than control) and 282 mg g^−1^ of carbohydrates (25% greater than control) were reached at the 5th and 10th culture days, respectively. These results are similar to those found by Wang et al. (2022) [46], where *Chlorella sorokiniana* (Chlorophyta) grow under sulfur limitation conditions by obtaining >50% of carbohydrates compared to control conditions. Another microalga, *Chlorococcum infusionum* (*Chlorococcum humicola*) (Chlorophyta), reached a higher concentration of carbohydrates when grown in low-sulfur conditions, up to 10%, in comparison to standard conditions [47].

According to the ANOVA–Tukey pairwise analysis results of carbohydrates in *C. reinhardtii* culture, it was determined that the control [Medium] and low nitrogen [N^−^] concentration conditions produced a higher carbohydrates content in all the experiments at the 15th day of culture (175 mg g^−1^) and at the 5th day of culture (163 mg g^−1^), respectively. In the case of the ANOVA–Tukey pairwise analysis of carbohydrates in *C. vulgaris* culture, it was determined that at low phosphorus [P^−^] concentration, a higher carbohydrates content was produced in all the experiments at the 5th day of culture (355 mg g^−1^). As it was possible to observe in the case of *C. reinhardtii*, a higher concentration was obtained in the control, a result which is given at the 15th day of culture, which leads us to consider that this microalga tends to accumulate a greater amount of carbohydrates over time. However, in the case of low nitrogen concentration [N^−^], a greater amount of carbohydrates can be obtained on the 5th day of culture. Therefore, if its production is sought, its harvest can be considered at this time.

In this way, it can be shown that the deprivation of these macroelements could enhance the production of carbohydrates in both microalgae; however, it is important to remark that each strain has a different behavior under these conditions. The increase in carbohydrates in microalgae biomass due to nutrients limitation conditions could be related to the fact that cells storing energy in the form of carbohydrates use less energy than they do by storing it in lipids (50% less ATP and 45% less NADPH in TAG synthesis process) [48]. Nitrogen and phosphorus limitation affects photosynthetic activity in microalgae by reducing the chlorophyll content and using absorbed energy capacity [49]. The accumulation of energy as carbohydrates guarantees the availability of energy for DNA replication and general cell metabolism [50], which is confirmed because under nitrogen limitations, microalgae increase their gene expression of starch-degrading enzymes [51].

Under phosphorus limitation conditions, the exchange of triose phosphate (glyceraldehyde-3-phosphate; product of photosynthetic pathway) could be reduced by the lack of cytoplasmic orthophosphate, generating an accumulation of precursors (e.g., glucose-6-phosphate) of carbohydrates production such as starch biosynthesis [52,53]. The sulfur deprivation in microalgae culture also could enhance carbohydrates biosynthesis by redirecting metabolic carbon flux to increase energy storage biomolecules production. Some enzymes (starch synthase and glycogen branching enzyme) related to carbohydrates synthesis could be significantly up-regulated under sulfur deprivation conditions, enhancing in this way the formation of amylose and amylopectin in microalgae cells [54].

### 2.3. Protein Content of Chlamydomonas reinhardtii and Chlorella vulgaris Grown with Different Nitrogen, Phosphorus, and Sulfur Concentrations

**Figure 3:**
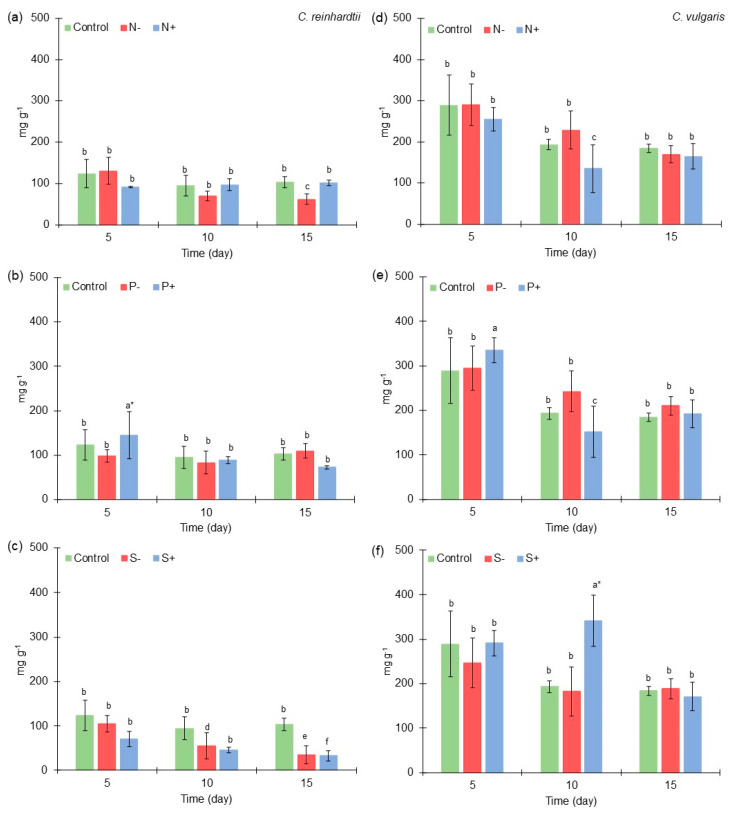
Protein content (mg g^−1^) of *Chlamydomonas reinhardtii* and *Chlorella vulgaris* grown at different nitrogen, phosphorus, and sulfur concentrations. (**a**) Protein content of *Chlamydomonas reinhardtii* grown at different nitrogen concentrations. (**b**) Protein content of *Chlamydomonas reinhardtii* grown at different phosphorus concentrations. (**c**) Protein content of *Chlamydomonas reinhardtii* grown at different sulfur concentrations. (**d**) Protein content of *Chlorella vulgaris* grown at different nitrogen concentrations. (**e**) Protein content of *Chlorella vulgaris* grown at different phosphorus concentrations. (**f**) Protein content of *Chlorella vulgaris* grown at different sulfur concentrations. The results of the ANOVA–Tukey pairwise analysis carried out for each microalga in all the variations in macroelements also could be observed by different groups expressed by letters (symbol * = express the treatment where a higher production was obtained throughout the experiment). All points were sampled by triplicate. The samples were taken every five days.

*C. reinhardtii* produced a higher concentration of proteins with medium macroelements concentration [Control] in most microalgae cultures, except in the cases of low nitrogen [N^−^] and high phosphorus [P^+^] concentration at the 5th culture day and low phosphorus [P^−^] concentration at the 15th culture day (Figure 3a–c). On the other hand, *C. vulgaris* exhibited an improved production of proteins under high macroelements concentrations in some microalgae cultures (Figure 3d–f).

At medium macroelements concentration [Control] in *C. reinhardtii*, 123 mg g^−1^, 94 mg g^−1^, and 103 mg g^−1^ were obtained at the 5th, 10th, and 15th culture days, respectively. These results are similar to those reported by Rosa et al. (2023) [55], where *C. reinhardtii* obtained approximately 50 mg g^−1^ to 150 mg g^−1^ of proteins under standard conditions. In a medium-concentration treatment, *C. vulgaris* obtained 289 mg g^−1^, 193 mg g^−1^, and 184 mg g^−1^ of proteins at the 5th, 10th, and 15th culture days, respectively. These results are in agreement with those reported by Ma et al., 2021 [56], who cultivated *C. sorokiniana* FZU60 under batch conditions and produced 400 mg g^−1^ of proteins after three days of culture.

At low nitrogen [N^−^] and high phosphorus [P^+^] concentration at the 5th culture day, *C. reinhardtii* obtained 130 mg g^−1^ (5% greater than medium concentration) and 145 mg g^−1^ (17% greater than medium concentration), respectively. These results are similar to those found by Kamalanathan et al. (2016) [57], where *C. reinhardtii* obtained a higher amount of protein in conditions without nitrogen in the first five days of culture. The increase in proteins at low nitrogen [N^−^] concentration could be related to the use of intracellular nitrogen stored in macromolecules such as chlorophyll. Similar to these results, *C. reinhardtii* culture under nitrogen limitation for the first cycles of cell division used the nitrogen found in the cells, and therefore, a slight increase (≤13%) in the protein content was observed in the culture [58].

Other authors have reported that *C. reinhardtii* at high phosphorus [P^+^] concentrations increased protein accumulation, which is in agreement with the results found in our work. Particularly, higher accumulation was reported of proteins involved in ribosome structure and synthesis, as well as in DNA and RNA metabolism [59]. Moreover, it has been demonstrated that this type of treatment [P^+^] leads to a reduction in the production of carbohydrates in the cell [59]. Similar to our study, the microalgae culture with a high phosphorus concentration had a 26% lower carbohydrates content than the control condition.

In the experiments on nitrogen variations, *C. vulgaris* obtained a greater amount of proteins at low nitrogen concentration [N^−^], where 228 mg g^−1^ (18% greater than medium [Control] concentration) was obtained at the 10th culture day. Under nitrogen limitation, microalgae can up-regulate proteins related to lipids (1-Acylglycerol-3-phosphate O-acyltransferase and Glycerol-3-phosphate O-acyltransferase) and carbohydrates (e.g., Citrate synthase, Aconitase, α-Ketoglutarate dehydrogenase, Succinic dehydrogenase, Fumarase) synthesis [60]. Also, at high phosphorus concentration [P^+^] of *C. vulgaris* culture, an increase in proteins content was observed, since it reached 335 mg g^−1^ (16% greater than medium concentration) at the 5th culture day. These results agree with those found in *C. reinhardtii* experiments.

Also, at low phosphorus concentration [P^−^] of *C. vulgaris* culture, a greater amount of proteins was obtained, but only on days 10 and 15 of the microalgae culture, with 242 mg g^−1^ (25% greater than control) and 210 mg g^−1^ of proteins (14% greater than control), respectively. In *Chlorella* sp. culture, it was found that phosphorus limitation could enhance protein production more than high phosphorus concentration [61], and these results are similar to those found in this study. This behavior could be related to microalgae’s capacity to preserve high levels of proteins involved in P’s storage as intracellular polyphosphate and its assimilation [59].

It is important to note that the variation in sulfur affects proteins content in *C. reinhardtii* in a negative way, especially in the high-concentration [S^+^] condition, since 70 mg g^−1^ (43% less than medium concentration), 45 mg g^−1^ (51% less than medium concentration), and 32 mg g^−1^ of proteins (>60% less than medium concentration) were obtained at the 5th, 10th, and 15th culture days, respectively. In addition, high sulfur concentration [S^+^] resulted in 341 mg g^−1^ (>50% greater than control) at the 10th culture day of *C. vulgaris* culture. Contrarily, *C. vulgaris* at low sulfur [S^−^] concentration produced a protein content decrease, which can be related to the affectation in biosynthesis of the sulfur amino acids such as cysteine and methionine [62]. Also, sulfur is a macronutrient related to nuclear transcripts encoding proteins involved in photosynthetic activity; therefore, its variation in culture medium has a negative impact on the protein content of the biomass [63].

From the ANOVA–Tukey pairwise analysis of protein content in *C. reinhardtii* culture, it was determined that at high phosphorus [P^+^] concentration, a higher protein content was produced in all the experiments at the 5th day of culture (145 mg g^−1^). On the other hand, from the ANOVA–Tukey pairwise analysis of protein in *C. vulgaris* culture, it was determined that at high sulfur [S^+^] and high phosphorus [P^+^] concentration was produced a higher protein content in all the experiments at the 10th day of culture (341 mg g^−1^) and at the 5th day of culture (335 mg g^−1^), respectively.

### 2.4. Lipid Content of Chlamydomonas reinhardtii and Chlorella vulgaris Grown with Different Nitrogen, Phosphorus, and Sulfur Concentrations

**Figure 4:**
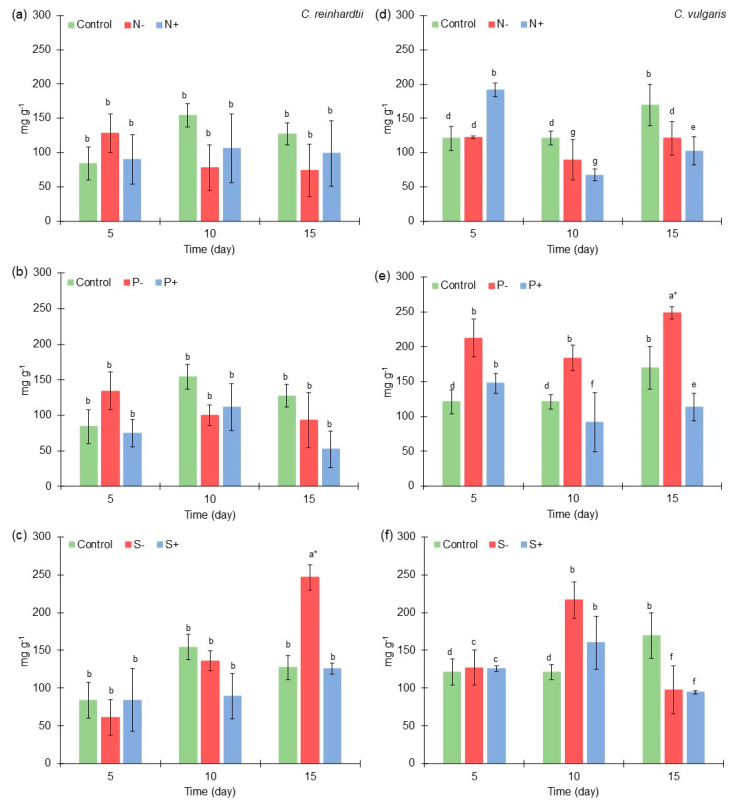
Lipid content (mg g^−1^) of *Chlamydomonas reinhardtii* and *Chlorella vulgaris* grown at different nitrogen, phosphorus, and sulfur concentrations. (**a**) Lipid content of *Chlamydomonas reinhardtii* grown at different nitrogen concentrations. (**b**) Lipid content of *Chlamydomonas reinhardtii* grown at different phosphorus concentrations. (**c**) Lipid content of *Chlamydomonas reinhardtii* grown at different sulfur concentrations. (**d**) Lipid content of *Chlorella vulgaris* grown at different nitrogen concentrations. (**e**) Lipid content of *Chlorella vulgaris* grown at different phosphorus concentrations. (**f**) Lipid content of *Chlorella vulgaris* grown at different sulfur concentrations. The results of the ANOVA–Tukey pairwise analysis carried out for each microalga in all the variations in macroelements also could be observed by different groups expressed by letters (symbol * = express the treatment where a higher production was obtained throughout the experiment). All points were sampled by triplicate. The samples were taken every five days.

*C. reinhardtii* at medium macroelements concentration [Control] produced 84 mg g^−1^, 154 mg g^−1^, and 127 mg g^−1^ of lipids at the 5th, 10th, and 15th culture days (Figure 4a–c), respectively. These results are similar to those obtained with *C. reinhardtii* cc849, for which 150 mg g^−1^ of lipids was reached at control conditions. Also, it was observed that lipids increased in microalgae biomass at least 50% after nitrogen deprivation [64]. This behavior was also found in this study, since low nitrogen [N^−^] concentration produced 128 mg g^−1^ (52% greater than medium [Control] concentration). On the other hand, *C. vulgaris* obtained an improved production of lipids with low macroelements concentrations in some microalgae cultures (Figure 4d–f). At medium macroelements concentration [Control], 121 mg g^−1^ and 169 mg g^−1^ were obtained at the 10th and 15th culture days, respectively. In other studies, *C. vulgaris* has had a lipid accumulation of 9 to 12% dry wt [65], which are similar results to those found here.

At low nitrogen concentration [N^−^], a slightly higher amount of lipids was obtained in *C. vulgaris*, but only on day 5 of the microalgae culture, when122 mg g^−1^ was reached. Similarly, *Graesiella emersonii* (formerly *Chlorella emersonii*) produced more lipids under nitrogen limitation conditions; however, a complete nitrogen deprivation reduced lipid production [37]. Hence, it is important to determine the optimal level of nitrogen limitation to improve lipid production in microalgae biomass depending on the microalgae strain. In this case, it is possible that with a concentration slightly higher than that considered in the low range, an increase in lipids could be observed.

The response of lipid production in microalgae culture under low-nitrogen conditions is related to the overexpression of genes involved in the TAG pathway. In *Tetradesmus bernardii* (Chlorophyta)*,* it was observed that genes encoding diacylglycerol acyltransferase (DGAT2 and DGAT1) were up-regulated under low nitrogen concentration, as well as acetyl-coenzyme A carboxylase [66]. Also, in this condition, an overexpression of genes related to the biosynthesis of palmitoyl-acyl carrier protein (β-ketoacyl-acyl-carrier-protein synthase [KASII]) could be observed in *Scenedesmus acutus* [67].

At low phosphorus [P^−^] concentration at the 5th culture day in *C. reinhardtii*, a higher production of lipids (134 mg g^−1^) was obtained (37% greater than medium concentration). Similar to *C. reinhardtii*, in the experiments on phosphorus variations, *C. vulgaris* obtained a greater amount of lipids under low phosphorus concentration [P^−^], reaching values of 212 mg g^−1^ (>50% greater than medium [Control] concentration), 183 mg g^−1^ (34% greater than medium concentration), and 248 mg g^−1^ (45% greater than medium concentration) at the 5th, 10th, and 15th culture days, respectively. This behavior is similar to that found in other microalgae such as *Scenedesmus* sp., which at lower phosphorus concentration produced an increase of approximately 50% in lipid content. The phosphorus limitation enhances the expression of genes related to fatty acid biosynthesis such as 3-ketoacyl-CoA synthase and 2-enoyl-CoA reductase [68]. In addition, lipids production could be associated with the low production of carbohydrates presented under the low phosphorus treatment [P^−^], since a similar carbohydrate content to that obtained by the control was observed on certain sampling days (Figure 2); contrarily, the production of lipids showed an evident increase regardless of the sampling day.

In the case of low sulfur [S^−^] concentration at the 15th culture day, 246 mg g^−1^ (>50% greater than control culture) was obtained in *C. reinhardtii* culture. *C. reinhardtii* CC-124 shows an increase in lipids during sulfur deprivation of approximately 35% [69]. These results are similar to those found by Gómez-De la Torre et al. (2023) [70], who reported a higher lipid content in sulfur and phosphorus limitation conditions, showing that the concentration of both macroelements in microalgae culture is highly relevant for the production of lipids. Also, 216 mg g^−1^ (>50% greater than control) was obtained under low concentration [S^−^] conditions at the 10th culture day in *C. vulgaris*. This behavior could be related to the overexpression of genes encoding acyltransferases related to TAG production such as phospholipid diacylglycerol acyltransferase (PDAT1) and diacylglycerol acyltransferase (DGTT1) [71]. In addition, it was determined that sulfur limitation could affect acetyl-CoA metabolomics flow, which in turn may increase the production of fatty acids and genes related to fatty acid desaturase and phosphatidic acid phosphatase [54].

From the ANOVA–Tukey pairwise analysis of lipids production in *C. reinhardtii* culture, it was determined that at low sulfur [S^−^] concentration, a higher lipids content was produced in all the experiments at the 15th day of culture (246 mg g^−1^). On the other hand, from the ANOVA–Tukey pairwise analysis of lipid production in *C. vulgaris* culture, it was determined that at low phosphorus [P^−^] concentration, a higher lipids content was produced in all the experiments at the 15th day of culture (248 mg g^−1^).

### 2.5. FAMEs Accumulation of Chlamydomonas reinhardtii and Chlorella vulgaris Grown with Different Nitrogen, Phosphorus, and Sulfur Concentrations

**Figure 5:**
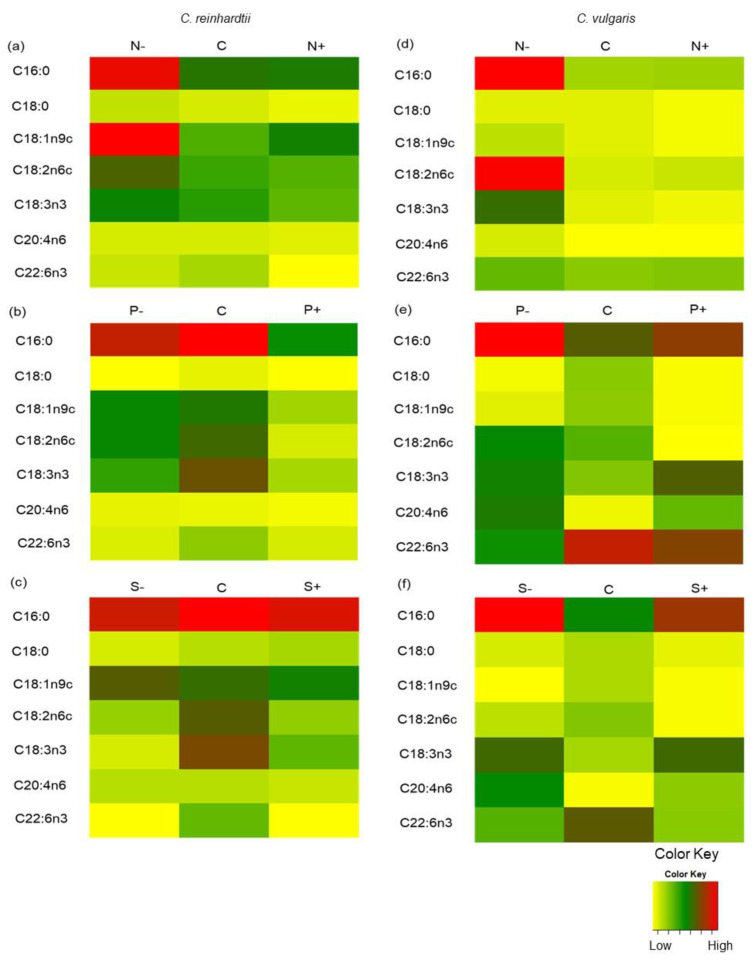
Heatmap of FAMEs (mg g^−1^) in *Chlamydomonas reinhardtii* and *Chlorella vulgaris* grown at different nitrogen, phosphorus, and sulfur concentrations. (**a**) FAMEs production of *Chlamydomonas reinhardtii* grown at different nitrogen concentrations. (**b**) FAMEs production of *Chlamydomonas reinhardtii* grown at different phosphorus concentrations. (**c**) FAMEs production of *Chlamydomonas reinhardtii* grown at different sulfur concentrations. (**d**) FAMEs production of *Chlorella vulgaris* grown at different nitrogen concentrations. (**e**) FAMEs production of *Chlorella vulgaris* grown at different phosphorus concentrations. (**f**) FAMEs production of *Chlorella vulgaris* grown at different sulfur concentrations. All points were sampled by triplicate. The samples were taken at 15th culture day.

*C. reinhardtii* in medium concentration of macronutrients [Control] and after 15 days of cultivation produced 20 ± 3 mg g^−1^ (31% of total fatty acids) of palmitic acid (C16:0), 12 ± 1.5 mg g^−1^ (17%_TFA_) of oleic acid (C18:1n9c), 13 ± 2.5 mg g^−1^ (16%_TFA_) of linoleic acid (C18:2n6c), and 15 ± 2.5 mg g^−1^ (18%_TFA_) of α-linoleic acid (C18:3n3) (Figure 5a–c). These results are similar to those found by Zheng et al., 2022 [72], where *C. reinhardtii* obtained 21%_TFA_ of palmitic acid, 9%_TFA_ of oleic acid, and 17%_TFA_ of linoleic acid.

At low nitrogen concentration [N^−^] in *C. reinhardtii* culture, 34 ± 8 mg g^−1^ [28%_TFA_] of palmitic acid, 36 ± 10 mg g^−1^ [27%_TFA_] of oleic acid, 23 ± 5 mg g^−1^ [19%_TFA_] of linoleic acid, and 19 ± 3 mg g^−1^ [16%_TFA_] of α-linoleic acid were produced at the 15th culture day, the highest result in the whole experiment with this microalga strain. In the literature, similar results can be found. For example, *Phaeodactylum tricornutum* produced a higher percentage of α-linoleic acid and palmitic acid under a low-nitrogen condition [73]. *Chlamydomonas malina* FT89.6 PG5 (Roscoff Culture Collection No. 2488) also showed the same behavior under low-nitrogen condition, since it accumulated a slightly higher amount of FAMEs [34].

On the other hand, *C. vulgaris* under low nitrogen concentration [N^−^] conditions produced 35 ± 12 mg g^−1^ of palmitic acid (5-fold greater than control), 6 ± 1.2 mg g^−1^ of oleic acid (2-fold greater than control), 34 ± 12 mg g^−1^ of linoleic acid (8-fold greater than control), 21 ± 8 mg g^−1^ of α-linoleic acid (6-fold greater than control), and 3.8 ± 0.75 mg g^−1^ of arachidonic acid (2-fold greater than control) at the 15th culture day (Figure 5d). Similar to *C. reinhardtii*, in this treatment, the highest result in the whole experiment was obtained. This behavior was observed in *Vischeria calaminaris* (formerly *Eustigmatos calaminaris*) culture, which, under low nitrogen concentration, produced higher C16 and C20 fatty acids [42]. These results suggest that decreasing the concentration of nitrogen might be a more feasible strategy to increase the percentage of fatty acids produced in microalgae, in comparison to strategies related to the concentration of phosphorus and sulfur.

In low phosphorus [P^−^] conditions, *C. reinhardtii* produced 5% more arachidonic acid (C20:4n6) than control conditions. This result is in agreement with other studies performed with *C. reinhardtii* culture, in which phosphorus stress generated longer-chain fatty acids (C18:0, C18:1n9c, C:20) compared to control conditions [69]. Similarly, under low phosphorus [P^−^] conditions in *C. vulgaris* culture (Figure 5e), higher concentrations of palmitic acid (44% greater), linoleic acid (42% greater), α-linoleic acid (>50% greater), and arachidonic acid (>50% greater) were observed in comparison to control. The increment can be related to the overexpression of genes involving in fatty acid biosynthesis (e.g., 3-oxoacyl-ACP reductase, 3-ketoacyl-CoA synthase), especially in the case of palmitic and arachidonic acid production [68]. *C. vulgaris* at high phosphorus concentration [P^+^], higher concentrations of palmitic (13% greater), α-linoleic acid (>50% greater), and arachidonic acid (>50% greater) were obtained compared to control.

Regarding sulfur treatments, low concentration [S^−^] resulted in 23%_TFA_ of oleic acid (10% more than control) in *C. reinhardtii* culture. Also, 2-fold greater control of arachidonic acid was obtained at low sulfur concentration. It has been demonstrated that high sulfur concentration causes negative effects on FAMEs production, except in the case of stearic acid, since it obtained >20% production compared to control. The increase in FAMEs concentration under low sulfur concentration could be related to the overexpression of genes related to acetyl-CoA carboxylase, 3-ketoacyl-ACP synthase, and 3-ketoacyl-ACP reductase production, which are involved in fatty acid synthesis [40].

*C. vulgaris* culture observed an increase in palmitic acid (>50% greater), linoleic acid (>50% greater), and arachidonic acid (>50% greater) compared to control at low sulfur concentration [S^−^] (Figure 5e). These results are similar to those found with *Chlorella vulgaris* under sulfur-deprived medium, where an increase in palmitic, oleic, and linoleic acid were observed [70]. In addition, at high sulfur concentration [S^+^], higher concentrations of palmitic acid (49% greater) and α-linoleic acid (>50% greater) were determined compared to the control.

## 3. Materials and Methods

### 3.1. Reagents and Equipment

The reagents used for culture medium preparation and to perform the carbohydrates, lipids, and FAMEs analysis were purchased from Sigma-Aldrich (Saint Louis, MO, USA). Lowry Protein Assay Kit was purchased from Thermo Fisher Scientific (Rockford, IL, USA). The spectrophotometric measurements were taken with an absorbance microplate reader from BGM Labtech (Fluorstar omega, 415-0470, Ortenberg, Germany).

### 3.2. Inoculum Culture Conditions

The strains *Chlamydomonas reinhardtii* and *Chlorella vulgaris* were purchased from UTEX (UTEX, Austin, TX, USA). BG11 culture medium was used to perform the experiments with both microalgae strains. The BG11 culture medium composition was as follows: NaNO_3_ 1.5 g L^−1^, K_2_HPO_4_ 40 mg L^−1^, CaCl_2_·2H_2_O 36 mg L^−1^, MgSO_4_·7H_2_O 75 mg L^−1^, Citric Acid·H_2_O 6 mg L^−1^, C_6_H_8_FeNO_7_ 6 mg L^−1^, Na_2_EDTA·2H_2_O 1 mg L^−1^, Na_2_CO_3_ 20 mg L^−1^, H_3_BO_3_ 2.86 mg L^−1^, MnCl_2_·4H_2_O 1.81 mg L^−1^, ZnSO_4_·7H_2_O 0.22 mg L^−1^, Na_2_MoO_4_·2H_2_O 0.39 mg L^−1^, CuSO_4_·5H_2_O 0.079 mg L^−1^, and Co(NO_3_)_2_·6H_2_O 0.04 mg L^−1^. The stock cultures were kept at 21 ± 1 °C with continuous light at 100 μmol photons m^−2^ s^−1^ and with 1.5 L m^−2^ min^−1^ of aeration (OPTIMA 4.5-Watt pump with an air filter of 0.20 µm).

### 3.3. Growth Measure of Chlamydomonas reinhardtii and Chlorella vulgaris with Different Nitrogen, Phosphorus, and Sulfur Concentrations

For each experiment, 100 mL *Chlamydomonas reinhardtii* and *Chlorella vulgaris* inocula, with an absorbance of 1 at the wavelength of 750 nm, were cultivated separately in lab glass bottles containing 900 mL of BG11. Before inoculation, the biomass was washed twice with bidistilled water. The cultures were maintained under the same conditions mentioned in the previous section. A factorial design was used for this research and applied to each experiment set (Table 1). Cell growth and biomass composition of *Chlamydomonas reinhardtii* and *Chlorella vulgaris* were evaluated under three different nitrogen, phosphorus, and sulfur concentrations. Nitrogen: low, 80 mg L^−1^ N; medium, 240 mg L^−1^ N; and high, 640 mg L^−1^ N. Phosphorus: low, 4 mg L^−1^ P; medium, 7 mg L^−1^ P; and high, 17 mg L^−1^ P. Sulfur: low, 8 mg L^−1^ S; medium, 20 mg L^−1^ S; and high 52 mg L^−1^ S. The foregoing values were obtained during 15 culture days. The medium concentration was taken as a control, since this is the standard concentration used in the BG11 medium.

Prior to the experiments, an equation of relationship between absorbance (750 nm) and dry weight (g L^−1^) of each strain was defined, as shown in Equations (1) and (2). Samples of 1 mL were obtained every day to assess the biomass growth curve by spectrophotometry. The dry weight was determined by filtering 10 mL of serial algal culture dilutions using pre-weighed Whatman GF/C glass microfiber filters. The filter holding the algal biomass was dried at 100 °C for 2 h. Then, filters with the algal biomass were cooled at room temperature inside a vacuum desiccator and weighed gravimetrically.

(1) $$C.reinhardtii\;g\;L^{-1}=3.9218\left({Abs}_{750}\right)-0.2085\;R^{2}=0.996$$

(2) $$C.vulgaris\;g\;L^{-1}=1.0444\left({Abs}_{750}\right)-0.1672\;R^{2}=0.991$$

### 3.4. Biomass Characterization of Chlamydomonas reinhardtii and Chlorella vulgaris Grown with Different Nitrogen, Phosphorus, and Sulfur Concentrations

The cultures of *Chlamydomonas reinhardtii* and *Chlorella vulgaris* were sampled by taking 15 mL of the culture on days 5, 10, and 15. Samples were stored at 4 °C until further analysis. To perform the carbohydrate, protein, and lipid assay, samples were centrifuged at 6000 rpm and 4 °C for 20 min. Then, the supernatant was discharged, and the biomass was washed twice and resuspended in distilled water.

### 3.5. Total Carbohydrates Content of Chlamydomonas reinhardtii and Chlorella vulgaris Grown with Different Nitrogen, Phosphorus, and Sulfur Concentrations

Carbohydrate content was quantified using the sulfuric acid–UV assay described by López-legarda et al. [74]. Briefly, 300 µL of the washed biomass was taken before adding 1 mL of concentrated sulfuric acid; then, the mixture was incubated in an ice bath for two minutes. Then, the absorbance was measured at 315 nm using a microplate reader from BGM Labtech. Finally, the absorbance readings were used to obtain the concentration of carbohydrates (mg L^−1^) by using a glucose standard curve.

### 3.6. Total Protein Content of Chlamydomonas reinhardtii and Chlorella vulgaris Grown with Different Nitrogen, Phosphorus, and Sulfur Concentrations

The Modified Lowry Protein Assay Kit was used to determine the total protein produced by *Chlamydomonas reinhardtii* and *Chlorella vulgaris*. Firstly, 1 mL of Lowry’s reagent was added to 200 µL of washed biomass solution. Secondly, the tube was vortexed. After 10 min, 100 µL of Folin reagent was added, and the mixture was vortexed again. After 30 min of reaction in the dark, the absorbance of the samples was measured at 750 nm in an absorbance microplate reader from BGM Labtech. Finally, the protein concentration was obtained using a BSA calibration curve.

### 3.7. Total Lipid Content of Chlamydomonas reinhardtii and Chlorella vulgaris Grown with Different Nitrogen, Phosphorus, and Sulfur Concentrations

The lipid content produced by *Chlamydomonas reinhardtii* and *Chlorella vulgaris* was quantified using the sulfo-phospho-vanillin assay described by Mishra et al. (2014) [75]. Briefly, 2 mL of sulfuric acid (98%) was added to 100 µL of the concentrated washed microalgae biomass (10:1). Then, it was heated for 10 min at 100 °C. Next, it was cooled in an ice bath for 5 min. After that, 5 mL of phospho-vanillin reagent was added, and the sample was incubated for 15 min at 37 °C. Finally, the absorbance was measured at 530 nm in an absorbance microplate reader from BGM Labtech. The lipid concentration was obtained using a canola oil calibration curve.

### 3.8. FAMEs of Chlamydomonas reinhardtii and Chlorella vulgaris Grown with Different Nitrogen, Phosphorus, and Sulfur Concentrations

Chloroform, methanol, and distilled water were added in ratios of 1:2:0.4 (*v*/*v*/*v*), respectively, into 150 mg of dried biomass. The resulting mixture was mixed for 30 s and then centrifuged at 4800 rpm for 10 min at 4 °C. After centrifugation, the lower layer was carefully recovered and filtered using a PTFE syringe filter (0.45 μm pore size, Thermo Fisher Scientific, Waltham, MA, USA). Then, it was transferred into a pre-weighed glass tube. The remaining chloroform was dried in a concentrator at 45 °C for 2 h at 15 mm Hg. The extracted lipids were gravimetrically weighted to estimate the total lipid content according to the Bligh and Dyer method with some modifications [76].

Fatty acids were determined through derivatization to fatty acid methyl esters (FAMEs) in closed vial by using (2 mL) of methanol at 7% with sulfuric acid using triundecanoin (C11:0) as internal standard. Mixture was heated at 80 °C for 1.5 h. Immediately after reaching room temperature, sample was extracted with 3 mL of hexane while manually shaking for 1 min. The upper phase formed was recovered, and the inferior phase was extracted again with 3 mL of hexane. Recovered phases were placed in a volumetric flask and brought to 10 mL with hexane.

Fatty acid profile was analyzed by a gas chromatograph with a mass spectrometer (Perkin Elmer, Clarus 600/560D, Bridgeport, CT, USA) by using an HP-88 capillary column (100 m, 0.25 mm × 0.20 µm). The carrier gas was helium at a constant flow of 1 mL/min. The initial oven temperature was 140 °C (held for 5 min), and it was increased by 4 °C/min until it reached the final temperature 240 °C (held for 15 min). Split ratio was 1:10, injector temperature was 265 °C, mass spectrometer analysis used EI ion source, electron energy was 70 eV, the temperature of source and interface were 210 °C, and range of *m*/*z* = 30–550. For FAMEs identification, the retention time of a typical chromatogram of the 37-component standard reference for FAME was used, and the quantification was realized used the standard internal method, using triundecanoin as the internal standard (AOAC Official Method 996.06).

### 3.9. Data Analysis

All data were analyzed by Minitab 21 (Minitab Inc., PA, USA). They were analyzed by an ANOVA with a Tukey pairwise comparison for each type of microalga (α = 0.05). The heatmaps for FAMEs expression were created with R-studio using ggplot2 package.

## 4. Conclusions

The results found in this study allow us to visualize the impact of three macronutrients’ (nitrogen, phosphorus, and sulfur) variation on the production of biomolecules of interest in the same microalgae strain (*Chlamydomonas reinhardtii* and *Chlorella vulgaris*). It was shown that some microalgae (*C. reinhardtii*) could produce more biomass under low nitrogen concentration (3.69 g L^−1^) than standard conditions. It was shown that low concentrations of nitrogen and phosphorus could enhance carbohydrates and lipids in microalgae culture. In addition, surprisingly, the impact that sulfur variation can have on the production of biomolecules in microalgae was found, especially in the case of lipids and proteins. Interestingly, it was determined that low concentration of nitrogen had a better effect on accumulation of some FAMEs (e.g., palmitic, oleic, and linoleic acid) in both microalgae. These results could be useful for increasing productivity of some biomolecules in large-scale microalgae cultures, especially in those intended for the production of FAMEs (C16-C:18).

## Figures and Tables

**Table 1:** Experimental design to evaluate the effect of the nutrient concentration (nitrogen, phosphorus, and sulfur) on growth behavior and biomass composition in *Chlamydomonas reinhardtii* and *Chlorella vulgaris* culture.

RunEst	Run	Blocks	Concentration	Nutrient
11	1	2	Low	S
17	2	2	High	S
18	3	2	High	P
12	4	2	Low	P
15	5	2	Med	P
16	6	2	High	N
13	7	2	Med	N
10	8	2	Low	N
14	9	2	Med	S
24	10	3	Med	P
20	11	3	Low	S
19	12	3	Low	N
22	13	3	Med	N
25	14	3	High	N
26	15	3	High	S
23	16	3	Med	S
21	17	3	Low	P
27	18	3	High	P
8	19	1	High	S
6	20	1	Med	P
3	21	1	Low	P
4	22	1	Med	N
1	23	1	Low	N
5	24	1	Med	S
7	25	1	High	N
2	26	1	Low	S
9	27	1	High	P

Nutrients: Nitrogen (Low [N^−^]: 80 mg L^−1^ N; Med [Control]: 240 mg L^−1^ N; High [N^+^]: 640 mg L^−1^ N). Phosphorus (Low [P^−^]: 4 mg L^−1^ P; Med [Control]: 7 mg L^−1^ P; High [P^+^]: 17 mg L^−1^ P). Sulfur (Low [S^−^]: 8 mg L^−1^ S; Med [Control]: 20mg L^−1^ S; High [S^+^]: 52 mg L^−1^ S). Factorial design has been carried out for each type of microalgae strain (*Chlamydomonas reinhardtii* and *Chlorella vulgaris*).

## Data Availability

All data related to this work is given in this manuscript.
